# Lip-reading and eye-gaze discrimination are functionally lateralized across the left and right posterior superior temporal sulci

**DOI:** 10.1093/cercor/bhag088

**Published:** 2026-06-24

**Authors:** Magdalena W Sliwinska, Theodoros Karapanagiotidis, Lara Nikel, Sara L Simone, Jonathan Smallwood, Elizabeth Jefferies, Andre Gouws, David Pitcher

**Affiliations:** School of Psychology, Liverpool John Moores University, James Parsons Building, 3 Byrom Street, Liverpool L3 3AF, United Kingdom; Department of Psychology, University of Sussex, Pevensey 1 Building, Falmer, North-South Road, Brighton BN1 9RH, United Kingdom; Nuffield Department of Clinical Neurosciences, University of Oxford, Level 6, West Wing, John Radcliffe Hospital, Headley Way, Oxford OX3 9DU, United Kingdom; School of Psychology, Liverpool John Moores University, James Parsons Building, 3 Byrom Street, Liverpool L3 3AF, United Kingdom; Department of Psychology, Queen’s University Ontario, 62 Arch Street, Kingston, ON K7L 3N6, Canada; Department of Psychology, University of York, Heslington, York YO10 5DD, United Kingdom; Department of Psychology, University of York, Heslington, York YO10 5DD, United Kingdom; Department of Psychology, University of York, Heslington, York YO10 5DD, United Kingdom

**Keywords:** eye-gaze, lip-reading, resting-state functional magnetic resonance imaging (rs-fMRI), third visual pathway, transcranial magnetic stimulation (TMS)

## Abstract

The posterior superior temporal sulcus (pSTS) processes information from the eyes and the mouth that support social perception. To investigate the laterality of how these mechanisms function, we performed three experiments on lip and eye-gaze discrimination. In Experiment 1, participants (*n* = 18) performed lip-position and eye-gaze discrimination tasks in static facial expressions while transcranial magnetic stimulation (TMS) was delivered over the left and right pSTS. Results showed a double dissociation in which disruption of the left pSTS impaired the lip-position task, while disruption of the right pSTS impaired the eye-gaze matching task. In Experiment 2, participants (*n* = 16) performed a lip-reading task using dynamic video clips of a speaker while TMS was delivered over the left and right pSTS. Task performance was impaired when TMS was delivered over the left pSTS only. In Experiment 3, participants (*n* = 256) underwent resting-state functional magnetic resonance imaging. Results demonstrated that the left pSTS exhibited greater connectivity to language processing brain areas in the left hemisphere. In contrast, the right pSTS exhibited greater connectivity to visual areas specialized for face processing and spatial attention processing. Our study suggests that lip and eye-gaze discrimination are preferentially lateralized across the bilateral pSTS.

## Introduction

Human social interactions depend on the ability to perceive and interpret actions and intentions of others, and to respond appropriately to a range of socially relevant cues (Frith and Frith 2012). These cues include facial expressions, eye-gaze direction, body movements, and the audiovisual integration of speech. The superior temporal sulcus (STS) plays a crucial role in this function by processing visual and auditory sensory information that underpins the understanding and interpretation of social cues (Puce et al. 1998; Allison et al. 2000; Hein and Knight 2008; Deen et al. 2015; Schobert et al. 2018). Neuroimaging studies show that the STS responds to facial expressions (Winston et al. 2004; Pitcher et al. 2020), eye-gaze (Calder et al. 2007), body movements (Grossman et al. 2010), mouth movements (Pelphrey et al. 2005), language (Binder et al. 1997), and audiovisual speech integration (Hocking and Price 2008). Studies of neuropsychological patients also demonstrate that damage to the STS impairs eye-gaze discrimination tasks (Akiyama et al. 2006a), alters the neural response to the McGurk effect (Baum et al. 2012), and the recognition of moving facial expressions (Sliwinska, Bearpark, et al. 2020; Prabhakar et al. 2025). The causal role of the STS in social perception has also been demonstrated using transcranial magnetic stimulation (TMS). TMS has been shown to impair eye-gaze discrimination (Pourtois et al. 2004), the McGurk effect (Beauchamp et al. 2010), and facial expression recognition (Pitcher 2014). However, despite substantial evidence implicating the STS in social cue processing, it remains unclear whether these functions are organized in a hemispherically lateralized manner and whether any such lateralization depends on the specific type of social information being processed (for example, eye-gaze versus lip movements). Addressing this question is particularly important given that many prior studies have targeted the STS in only one hemisphere, limiting direct comparisons of left and right hemisphere contributions.

One source of evidence for hemispheric asymmetry in the STS comes from studies of facial expression recognition and eye-gaze perception. Functional magnetic resonance imaging (fMRI) studies of visual face processing frequently report stronger responses in the right posterior STS (pSTS), consistent with a degree of right lateralization (Pitcher et al. 2007; Pitcher et al. 2011; Nikel et al. 2022; Lesinger et al. 2023; Kucuk et al. 2024), although the robustness of this asymmetry remains debated (Thome et al. 2022). Right-lateralized involvement of the pSTS has also been observed in processing eye-gaze direction. fMRI studies typically report bilateral pSTS activation during eye-gaze discrimination (Hoffman and Haxby 2000; Sato et al. 2008) but also suggest a right hemisphere bias, particularly for averted gaze (Engell and Haxby 2007). This is consistent with neuropsychological evidence from patients with right superior temporal gyrus lesions, who show selective impairments in gaze perception (Akiyama et al. 2006a; Akiyama et al. 2006b). Furthermore, TMS applied to the right pSTS (but not the left STS) has been shown to increase reaction times during eye-gaze discrimination tasks (Pourtois et al. 2004). Together, these findings suggest a preferential, though not exclusive, role of the right pSTS in processing socially relevant facial information, encompassing both facial expressions and eye-gaze cues.

In contrast to the relatively consistent right hemisphere bias reported for facial expressions and eye gaze, evidence for hemispheric specialization in the processing of visual speech cues is more mixed. A substantial literature demonstrates that visual information from the mouth and lips enhances speech comprehension (McGurk and MacDonald 1976; Walker et al. 1995; Alsius et al. 2005), reflecting audiovisual integration mechanisms that are strongly associated with pSTS function (Calvert et al. 1997; Hall et al. 2005; Lee and Noppeney 2011). However, it remains debated whether audiovisual integration per se elicits greater pSTS activation than unimodal stimulation when attentional and task demands are controlled (Hocking and Price 2008). Both static and dynamic facial speech stimuli robustly activate the pSTS bilaterally (Calvert and Campbell 2003), raising questions about the extent to which speech related visual processing is hemispherically specialized.

Some studies suggest that the hemispheric organization of the pSTS may depend on the social cue being processed. For example, De Winter (2015) reported left lateralized pSTS responses to speech accompanied facial expressions, whereas non-speech expressions preferentially engaged the right pSTS. Similarly, TMS disruption of the left pSTS has been shown to impair perception of the McGurk effect, implicating left hemisphere involvement in audiovisual speech integration (Beauchamp et al. 2010). Critically, however, these studies did not include stimulation or direct comparison of the right pSTS, preventing firm conclusions regarding hemispheric specialization.

Taken together, existing research suggests a preferential role of the right pSTS in processing visually conveyed social cues related to faces and eye gaze, whereas speech-related visual information may recruit the pSTS more bilaterally or with a left hemisphere bias. However, because most prior studies have examined the STS in only one hemisphere, it remains unresolved whether social cue processing within the STS is intrinsically lateralized and whether any hemispheric differences depend on the specific type of social information being processed. Resolving this issue requires a direct, within study comparison of left and right pSTS contributions across different classes of social cues.

A series of our TMS studies has demonstrated a causal role for both left and right pSTS in facial expression recognition (Pitcher 2014; Sliwinska and Pitcher 2018; Sliwinska, Elson, and Pitcher 2020). Using a delayed match-to-sample task with static facial stimuli, these studies showed that disruption of the left and right pSTS impairs performance, with evidence for right-hemisphere dominance (Sliwinska and Pitcher 2018; Sliwinska, Elson, and Pitcher 2020). However, these studies did not address whether hemispheric differences within pSTS depend on the type of facial cue being processed. Crucially, different facial features convey distinct kinds of social information (Allison et al. 2000; Haxby et al. 2000). Eye-gaze signals are closely linked to visuospatial attention, intention inference, and social orienting mechanisms such as gaze cueing (Frischen et al. 2007). These processes are strongly associated with right-lateralized attentional systems, particularly the ventral attention network involved in stimulus-driven reorienting (Corbetta and Shulman 2002). In contrast, mouth movements provide critical visual input for speech perception and audiovisual integration, as demonstrated behaviorally and at the neural level by classic and contemporary studies of visual speech influences on auditory processing (McGurk and MacDonald 1976; Calvert et al. 1997). These speech-related processes are strongly associated with left-dominant language networks involving temporal and fronto-temporal cortices (Price 2000; Hickok and Poeppel 2007). Whether these functional distinctions are reflected in causal hemispheric specialization within pSTS remains an open question.

The present study was designed as a direct extension of our previous TMS investigations of bilateral pSTS. In Experiment 1, we used the same behavioral task and stimuli previously shown to be sensitive to facial expression disruption during pSTS stimulation (Pitcher 2014; Sliwinska and Pitcher 2018) but manipulated whether task-relevant information was derived from eye-gaze direction or lip position. This allowed us to test whether causal contributions of left and right pSTS depend on the facial feature supporting task performance while maintaining methodological continuity with earlier work. In Experiment 2, we tested whether left-hemisphere pSTS involvement in mouth-based judgments generalizes to dynamic, speech-related lip movements during a lip-reading task using non-emotional stimuli. Finally, in Experiment 3, we used resting-state functional MRI (rs-fMRI) to determine whether the causal dissociations observed in the TMS experiments correspond to distinct large-scale functional connectivity profiles of left and right pSTS. Together, these experiments test the hypothesis that hemispheric specialization within pSTS reflects differential engagement with speech-related versus visuospatial social information.

## Method

### Experiment 1: TMS effects on lip-position and eye-gaze

#### Participants

Eighteen right-handed participants (10 women and 8 men, aged 19 to 25 years old, M = 21 years and SD = 1.38) were recruited in this study. All participants were neurologically healthy with normal or corrected-to-normal vision. A Participant Information Sheet explaining the experimental procedures was provided to the participants prior to testing. Informed consent was obtained from all participants after the experimental procedures were explained. All participants were paid for their time. The study was approved by the York Neuroimaging Centre Research Ethics Committee at the University of York (P1405). All participants tolerated TMS stimulation with no significant discomfort and none withdrew from the study.

#### Experimental procedures

Each participant completed two testing sessions performed on different days. In the first session, participants completed a 40-min fMRI session designed to functionally localize the face-selective areas in the bilateral pSTS. In the second session, participants completed a 1-h TMS session during which they performed the eye-gaze matching or lip-position matching behavioral tasks while TMS was delivered over the functionally localized right or left pSTS. Participants also completed both tasks without TMS which acted as the stimulation control condition. No TMS control condition was chosen over an active control site (eg Vertex) to ensure a safe amount of stimulation during a single session (Wassermann 1998; Rossi et al. 2009).

### Session 1: fMRI functional localization of TMS target sites

#### Stimuli

Stimuli comprised short movie clips of dynamic faces or objects. Each movie clip lasted 3 s and presented a face or an object. Each stimulus category (ie faces or objects) included 60 movie clips in which distinct faces or objects appeared several times. Dynamic faces were used to increase the likelihood of identifying face-selective areas in pSTS, as this region responds more strongly to dynamic than to static stimuli, with spatial overlap in activations for both stimulus types (Pitcher et al. 2011). These stimuli were successful in localizing bilateral face-selective pSTS sites in our previous studies (Pitcher et al. 2017; Pitcher et al. 2019; Sliwinska, Elson, and Pitcher 2020; Sliwinska et al. 2021).

#### Procedure

Participants were scanned with fMRI while viewing two block-design runs during which they were asked to watch movie clips of dynamic faces or objects. Each run consisted of 10 blocks, half of which included faces and another half objects. Within each block, six 3 s movie clips of faces or objects were presented. Each block lasted 18 s; therefore, each run lasted 234 s, and 18 s rest blocks occurred at the beginning, middle, and end of each run. A high-resolution structural brain scan was also acquired to register the functional data for each participant onto their own brain anatomy.

#### Data collection

Imaging data were collected using a 3T Siemens Magnetom Prisma MRI scanner (Siemens Healthcare, Erlangen, Germany) at the York Neuroimaging Centre. Functional localization images from the whole brain were acquired using a 20-channel phased array head coil tuned to 123.3 MHz and a gradient-echo EPI sequence (60 interleaved slices; repetition time [TR] = 2,000 msec; echo time [TE] = 30 msec; flip angle = 80^o^; voxel size = 3 × 3 × 3 mm; matrix size = 80 × 80; field of view [FOV] = 240 × 240 mm; total number of volumes per run = 117). Slices were aligned with the anterior to posterior commissure. Structural images were acquired using a high-resolution T1-weighted MPRAGE sequence (176 interleaved slices; TR = 2,300 msec; TE = 2.26 msec; flip angle = 8^o^; voxel size = 1 × 1 × 1 mm; matrix size = 256 × 256; FOV = 256 × 256 mm).

#### Data analysis

Functional localization data were analyzed for each participant at the single-subject level in individual native space using fMRI Expert Analysis Tool (FEAT) within the FMRIB (v6.0) Software Library (www.fmrib.ox.ac.uk/fsl). First- and second-level analyses were performed to combine the functional data from all the runs for each individual participant. In the first-level analysis, as part of pre-statistical processing, single participant functional localization images underwent extraction of non-brain structures using the Brain Extraction Tool (BET). In addition, interleaved slice timing correction, MCFLIRT motion correction, spatial smoothing using a 5 mm full-width half-maximum Gaussian kernel, high-pass temporal filtering, and pre-whitening were applied. To obtain participant-specific patterns of activation, the preprocessed functional images were entered into a general linear model (GLM), with two independent predictors: faces (predictor 1) and objects (predictor 2). The model was convolved using a double-gamma hemodynamic response function (HRF) to generate the main regressors, and temporal derivatives for each condition were also included.

Face-selective areas in the right and left pSTS were identified using a contrast of faces greater than objects. First-level functional results were registered to the anatomical scan using a 6 degree-of-freedom affine registration. All analyses were conducted at the whole-brain level. In the second-level analyses, the first-level results for all individual runs of the functional localizer were averaged using fixed-effects and single group average model. The results were thresholded at the whole-brain level using cluster-based Gaussian random field theory, with a cluster-forming threshold of z > 3.1 and Family-Wise Error (FWE)-corrected cluster significance level of *P* < 0.05.

### Session 2: TMS and behavioral testing

#### Stimuli

Experimental stimuli consisted of 36 static images taken from the set of Ekman and Friesen (1976). Each image depicted a face making a facial expression. Faces of six female models (C, MF, MO, NR, SW, and PF) were used and each model expressed six different emotions: happy, sad, fear, surprise, disgust, and anger. These stimuli have been used in prior TMS experiments of the pSTS (Pitcher 2014; Sliwinska and Pitcher 2018; Sliwinska, Elson, and Pitcher 2020). Each image was cropped to the same contour to remove the models’ hair and neck, leaving only their faces. The stimuli were paired into 72 trials in each task. Within each trial, the two faces always had different identities, and within each run, all six expressions were presented equally often.

**Figure 1:**
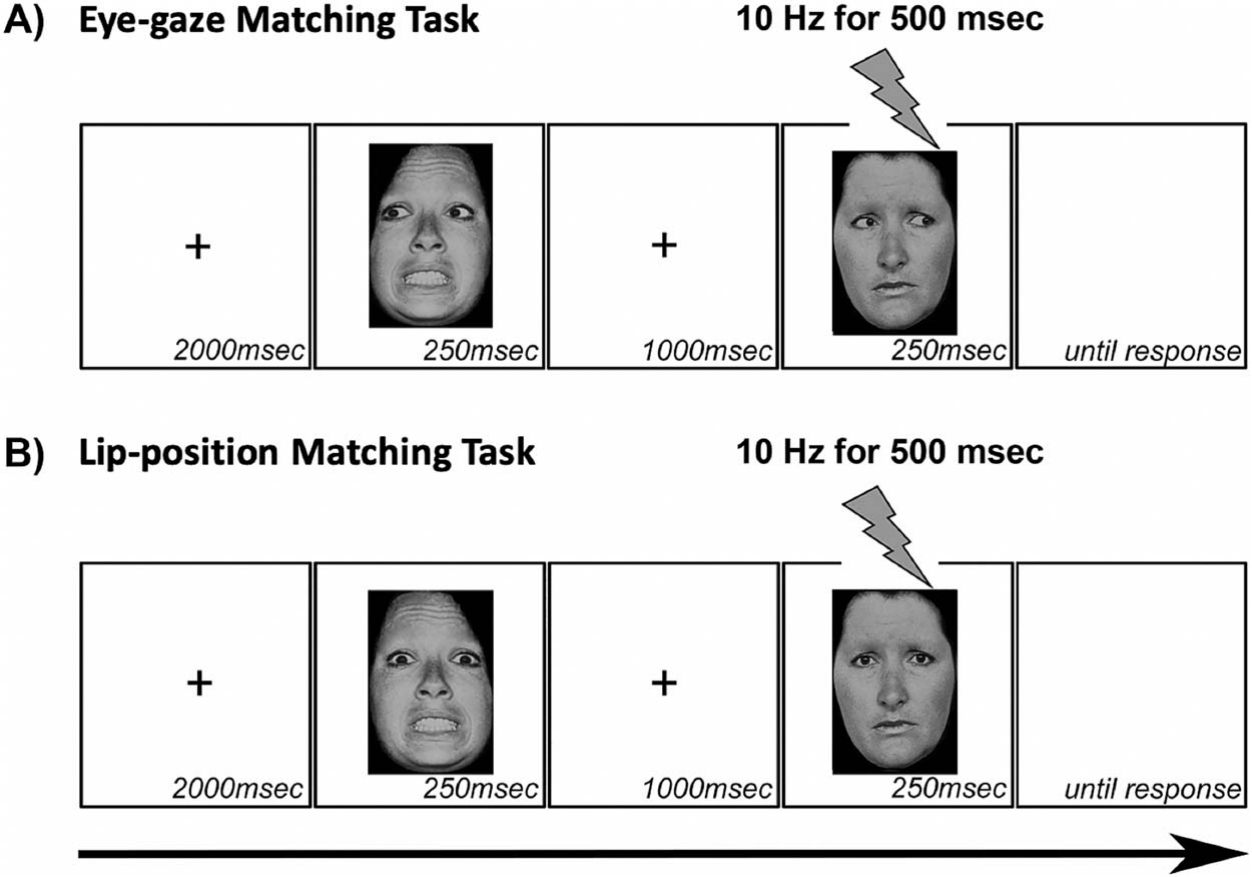
An example of a single trial in the A) eye-gaze matching task and B) lip-position matching task. Both examples are “no” trials.

For the eye-gaze matching task, the original images were manipulated using Adobe Photoshop CC (v2019; Adobe Inc., California, USA) to create five different eye-gaze conditions: (i) looking straight ahead (image unchanged); (ii) looking to the near left (iris and pupil were moved approximately 45 degrees to the left in both eyes); (iii) looking to the near right (iris and pupil were moved approximately 45 degrees to the right in both eyes); (iv) looking to the far left (iris and pupil were moved maximally to the left side of the eye in both eyes); (v) looking to the far right (iris and pupil were moved maximally to the right side of the eye in both eyes). Within a run, in half of the trials, the two faces looked in the same direction (eg both slightly to the right), whereas in the other half of the trials, each face looked in a different direction (eg one slightly to the right and the other slightly to the left). For the lip-position matching task, no image modifications were performed and original images in which actors look straight ahead were used. Within a run, half of the trials presented two faces with the same lip position (ie both lips closed or open), whereas in the other half of the trials, the lips position differed across the two faces (ie closed for one face and opened for the other). Figure 1 shows an example trial for each task.

#### Procedure

TMS behavioral data were acquired in a single session during which participants performed a delayed match-to-sample computer-based eye-gaze recognition task and lip-position recognition task. This task was adapted from a facial expression recognition task used in our previous studies in which TMS to the right and left pSTS robustly affected task performance (Pitcher 2014; Sliwinska and Pitcher 2018; Sliwinska, Elson, and Pitcher 2020). It is important to note that in this study, emotional expression was irrelevant to the task and never required for task performance. This task was used intentionally to ensure continuity and direct comparison with our previous TMS studies of bilateral pSTS, which used the same faces, timing and task structure to establish the causal involvements of left and right pSTS in facial expression processing. As in those previous studies, during each trial, participants were presented with two face images displayed sequentially (see Fig. 1). However, in the eye-gaze matching task, participants were asked to focus on the actors’ eyes and judge whether the actors were looking in the same direction or not. In the lip-position matching task, participants were asked to focus on the actors’ lips and judge whether both actors had their lips in the same position (ie both closed or both opened). Instructing participants to attend to either lip position or eye-gaze direction in the emotional facial stimuli enabled further investigation of the function that left and right pSTS play in the facial expression recognition task. Each face image was presented for 250 msec. Each image was preceded by a fixation cross for 2,000 msec and followed by a fixation cross for 1,000 msec. Each task trial ended with a blank white screen which remained until the participant responded.

During the testing session, participants completed three runs of each task. Each run of 72 trials lasted approximately 7 min. Each run consisted of the same trials that were randomized every time. TMS was delivered on each trial in conditions involving TMS. Runs for each task were completed under three different stimulation conditions: (i) TMS delivered to the right pSTS (stimulation condition 1), (ii) TMS delivered to the left pSTS (stimulation condition 2), or (iii) no TMS was delivered (stimulation condition 3). The third (no TMS) condition acted as a pure behavioral control for TMS effects occurring in the first and second stimulation conditions. The order of stimulation conditions was counterbalanced across participants.

For stimulation, online repetitive TMS (rTMS) was delivered as a train of 5 pulses at a frequency of 10 Hz (ie 1 pulse every 100 msec) for a duration of 500 msec at a fixed intensity of 60% of the maximum stimulator output. The fixed intensity value was used based on our previous studies (Pitcher et al. 2007; Pitcher et al. 2008; Pitcher et al. 2009; Sliwinska et al. 2021). Stimulation started at the onset of the second image to maximize the disruptive effect of eye-gaze or lip-position recognition.

#### TMS application

TMS was delivered using a Magstim Rapid2 stimulator and a Magstim coated Alpha Flat 50 mm diameter figure-of-eight coil (Magstim, Carmarthenshire, UK). The stimulation parameters were within established international safety limits (Wassermann 1998; Rossi et al. 2009). The TMS coil was held against the participant’s head by the experimenter who manually controlled its position throughout testing. All stimulation target sites identified from each participant’s functional localization data and were marked on their structural scan using the Brainsight frameless stereotaxic system (Rogue Research, Montreal, Canada). During testing, a Polaris Vicra infrared camera (Northern Digital, Waterloo, ON, Canada) was used in conjunction with the Brainsight to register the participant’s head to their structural scan for accurate stimulation targeting throughout the experiment. All participants wore earplugs in both ears to attenuate the sound of the coil discharge and prevent ear damage (Counter et al. 1991). In some participants, stimulation affected the peripheral jaw muscle and produced a small jaw twitch, but all participants found this sensation comfortable and non-distracting.

#### Data analysis

Performance reaction times (RTs) and accuracy were analyzed using IBM SPSS Statistics (v26.0) in a 2 × 3 repeated measures ANOVA, with Task (eye-gaze matching task and lip-position matching task) and Stimulation (rTMS to right pSTS, rTMS to left pSTS, and no TMS) as independent factors. Paired two-tailed t-tests (with Bonferroni correction for multiple comparisons) were used to further characterize significant main effects and interaction from the ANOVA.

#### Results

**Figure 2:**
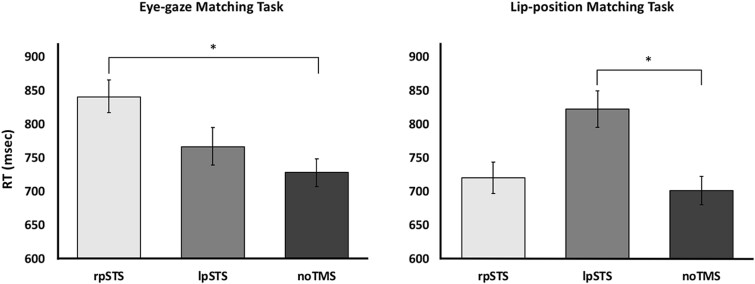
Group mean RTs for the eye-gaze matching task (left panel) and lip-position matching task (right panel) under three different stimulation conditions: 1) rTMS to the right pSTS (rpSTS, light gray bars); 2) rTMS to the left pSTS (lpSTS, dark gray bars); and 3) no TMS (black bars). Error bars represent “SEM” corrected for repeated measures. ^*^P < 0.008 corrected with Bonferroni correction for multiple comparisons.

Results for the group mean RTs are presented in Fig. 2. Most importantly, there was a significant two-way interaction between Task (eye-gaze matching task and lip-position matching task) and Stimulation (right pSTS, left pSTS, and no TMS) (F(2, 34) = 6.28; *P* = 0.005; partial ɲ^2^ = 0.27). T-tests revealed that in the eye-gaze matching task, when contrasted to the no TMS condition (729 msec; SD = 220 msec), the participants were significantly (t(17) = 3.15; *P* = 0.006; *d* = 0.55) slower when rTMS was applied to the right pSTS (841 msec; SD = 188 msec) while rTMS had no significant (t(17) = 1.20; *P* = 0.25; *d* = 0.17) effect on RTs when applied to the left pSTS (767 msec; SD = 236 msec). The opposite pattern of results was observed in the lip-position matching task, where in contrast to the no TMS condition (702 msec; SD = 212 msec), the participants were significantly (t(17) = 3.11; *P* = 0.006; *d* = 0.63) slower when rTMS was applied to the left pSTS (823 msec; SD = 167 msec) while rTMS had no significant (t(17) = 0.89; *P* = 0.39; *d* = 0.09) effect on RTs when applied to the right pSTS (721 msec; SD = 218 msec). The difference between the right and left pSTS in the eye-gaze matching task (t(17) = 1.68, *P* = 0.11, *d* = 0.35) and in the lip-position matching task (t(17) = 2.41, *P* = 0.03, *d* = 0.53) did not reach significance.

The main effect of Task (F(1, 17) = 1.52; *P* = 0.23; partial ɲ^2^ = 0.08) was not significant, with t-tests showing no significant (t(53) = 1.30; *P* = 0.20; *d* = 0.14) difference in RTs between the eye-gaze matching task (779 msec; SD = 216 msec) and the lip-position matching task (749 msec; SD = 204 msec). The main effect of Stimulation (F(2, 34) = 5.03; *P* = 0.01; partial ɲ^2^ = 0.23) was significant, with t-tests showing significantly (both t-tests: t(35) > 2.98; *P* < 0.005; *d >* 0.31) slower RTs when rTMS was applied to the right pSTS (781 msec; SD = 210 msec) or the left pSTS (795 msec; SD = 203 msec) in contrast to no TMS (716 msec; SD = 213 msec) condition. However, there was no significant (t(35) = 0.41; *P* = 0.69; *d* = 0.07) difference in RTs between right (781 msec) and left (795 msec) pSTS stimulation conditions.

The group mean accuracy results did not demonstrate significant effects of TMS on any task. The main effect of Stimulation (F(2, 34) = 0.79; *P* = 0.46; partial ɲ^2^ = 0.04) and a two-way interaction between Task (eye-gaze matching task and lip-position matching task) and Stimulation (right pSTS, left pSTS, and no TMS) (F(2, 34) = 1.39; *P* = 0.26; partial ɲ^2^ = 0.08) were not significant. Only, the main effect of Task was significant (F(1, 17) = 17.08; *P* < 0.001; partial ɲ^2^ = 0.50), with t-tests showing that performance in the eye-gaze matching task (95%; SD = 3%) was significantly (t(53) = 5.82; *P* < 0.001; *d* = 0.97) greater than performance in the lip-position matching task (91%; SD = 5%).

### Experiment 2: TMS effects on lip-reading

#### Participants

Sixteen right-handed participants (10 women and 6 men; aged 19 to 30 years old, M = 23 years and SD = 3) were tested in this study. All participants were neurologically healthy with normal or corrected-to-normal vision. A Participant Information Sheet explaining the experimental procedures was provided to the participants prior to testing. Informed consent was obtained from all participants after the experimental procedures were explained. All participants were paid for their time. The study was approved by the York Neuroimaging Centre Research Ethics Committee at the University of York (P1405). All participants tolerated TMS stimulation with no significant discomfort and none withdrew from the study.

#### Experimental procedures

Each participant completed two testing sessions performed on different days. In the first session, participants completed our functional localization of the face-selective areas in the bilateral pSTS, as described in the experiment 1. In the second session, participants completed a 1-h TMS session during which they performed the behavioral lip-reading tasks while TMS was delivered over the functionally localized right pSTS, left pSTS, or Vertex.

#### Stimuli

Experimental stimuli consisted of 135 dynamic color videos of a speaker saying a word. Each video presented a different word. For the purposes of this study, videos were filmed without sound. The same female native English speaker was used in all the videos. In each video, only the speaker’s head was visible on a black background. Each video was 2 s long. Words presented by the speaker were short, consisting of a single (eg “meal”) or two syllables (eg “wrestle”). The videos were used to create a task block consisting of 90-word pairs. Forty-five videos were used to create “yes” trials in which the same word was pronounced (eg “nurse—nurse”) while the remaining 90 videos were used to create “no” trials in which two different words were presented (eg “fear—hear”). To increase difficulty, the words were minimal pairs differing by only one phoneme (eg “hear—fear or green—grin”).

#### Procedure

**Figure 3:**
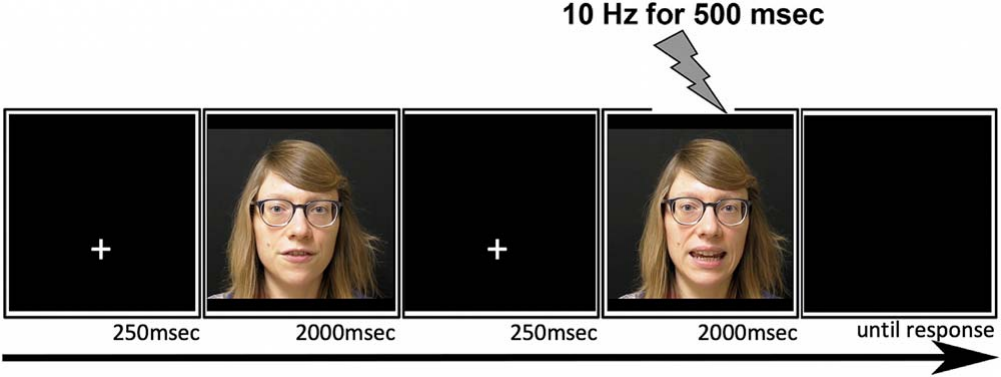
An example of a single task trial. This is a static representation of the dynamic video stimuli. Here, presenting the example of a “no trial” “fear—hear”.

The behavioral data were acquired in a single session during which participants performed a delayed match-to-sample computer-based word matching task in which they judged whether two words mouthed subsequently without any sound were the same or not. This task was created for the purposes of this study to test the involvement of the face-selective pSTS in lip-reading/processing of speech-related lip movements, in the absence of emotional facial stimuli and with mouth movements directly linked to speech perception. During each trial, participants were presented with two videos displayed subsequently (see Fig. 3). Before each video, a fixation cross was displayed for 250 msec at the lower part of the mid-screen which corresponded to the location of the speaker’s lips. Each task trial ended with a blank black screen displayed until the participant provided a response. Participants could respond at any time from the onset of the second video.

During the testing session, participants completed four runs of the task. Each run of 90 trials lasted approximately 6 min. The same trials appeared in each run, and their order was randomized across runs. TMS was delivered on each trial. Runs for each task were completed under three different stimulation conditions: (i) TMS delivered to the left pSTS (stimulation condition 1), (ii) TMS delivered to the right pSTS (stimulation condition 2), or (iii) Vertex (stimulation condition 3). The third stimulation condition acted as control site for nonspecific effects of TMS. The order of stimulation conditions was pseudorandomized across participants. At the beginning of each session, participants completed a training run of the task without TMS which was not included in the analysis. For stimulation, an identical protocol and stimulation apparatus were used as in the previous study. The only modification was that online rTMS (a train of 5 pulses at a frequency of 10 Hz and a fixed intensity of 60%) was synchronized with the onset of the speaker’s lip movements in the second video (see Fig. 3).

#### Data analysis

Performance RTs were analyzed using IBM SPSS Statistics (v29.0) with a one-way repeated-measures ANOVA, with Stimulation (rTMS to left pSTS, rTMS to right pSTS, and Vertex) as independent factors. Paired two-tailed t-tests (with Bonferroni correction for multiple comparisons) were used to further characterize the significant effect from the ANOVA. Performance accuracy was analyzed using a Friedman test for a nonparametric data set.

#### Results

**Figure 4:**
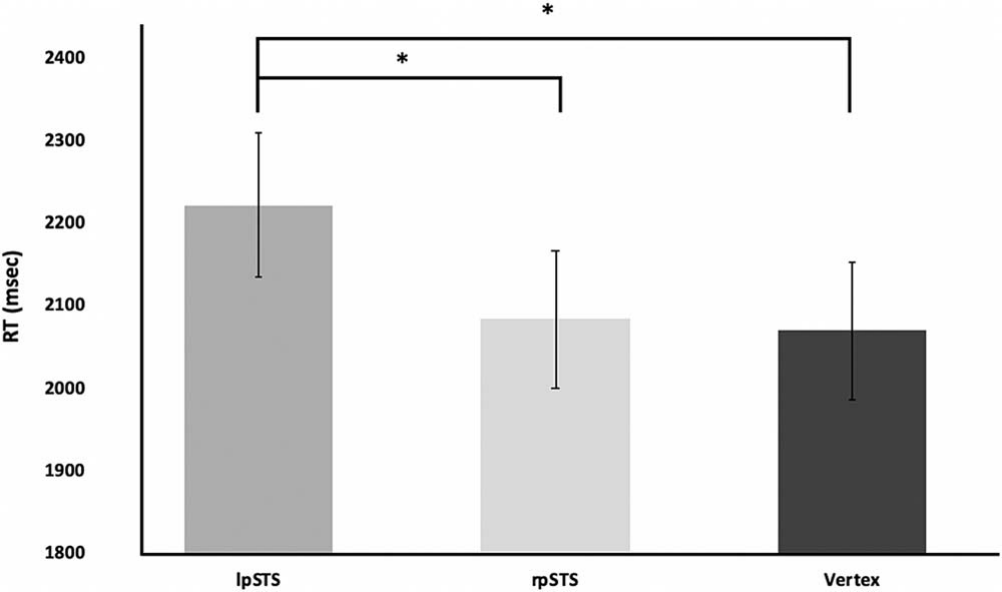
Group mean RTs for the lip-reading task under three different stimulation conditions: 1) rTMS to the left pSTS (lpSTS, dark gray bar); rTMS to the right pSTS (rpSTS, light gray bar); and 3) vertex (black bar). Error bars represent “SEM” corrected for repeated measures. ^*^P < 0.01 corrected with Bonferroni correction for multiple comparisons.

Results for the group mean RTs are presented in Fig. 4. One-way repeated measures ANOVA revealed a significant main effect of Stimulation (F(2, 30) = 8.44, *P* = 0.001; partial ɲ^2^ = 0.36), t-tests showing significantly slower reaction times when stimulation was applied to left pSTS (2,221 msec; SD = 350 msec) than right pSTS (2,083 msec; SD = 334 msec; t(15) = 3.07, *P* = 0.008, *d* = 0.077) or Vertex (t(15) = 3.44, *P* = 0.004, *d* = 0.86). There was no significant difference in reaction times when right pSTS (t(15) = 0.42, *P* = 0.68, *d* = 0.10) was compared to Vertex (2,070 msec; SD = 331 msec). For the accuracy data, Friedman test did not show a significant main effect of Stimulation (χ^2^(2) = 3.83, *P* = 0.15).

### Experiment 3: fMRI resting-state

#### Participants

Two hundred and seventy-seven healthy participants were recruited from the University of York. A Participant Information Sheet explaining the experimental procedures was provided to the participants prior to testing. Informed consent was obtained from all participants after the experimental procedures were explained. The study was approved by the York Neuroimaging Centre Ethics Committee (P1282/P1291), and all research was performed in accordance with relevant guidelines and regulations. All participants were paid for their time. Twenty-one participants were excluded from analyses, 1 due to technical issues during the neuroimaging data acquisition and 20 for excessive movement during the fMRI scan (mean framewise displacement >0.3 mm and/or more than 15% of their data affected by motion), resulting in a final cohort of *n* = 256 (169 women and 87 men; M = 21 years, SD = 2). The data from those participants have also been used to explore individual differences in the neural basis of semantic cognition and mind-wandering (Karapanagiotidis et al. 2020; Mckeown et al. 2020; Shao et al. 2022; Krieger-Redwood et al. 2025).

#### MRI data acquisition

MRI data were acquired on a GE 3 Tesla Signa Excite HD x MRI scanner, equipped with an eight-channel phased array head coil at the York Neuroimaging Centre, University of York. For each participant, we acquired a sagittal isotropic 3D fast spoiled gradient-recalled echo T1-weighted structural scan (TR = 7.8 msec, TE = minimum full, flip angle = 20^o^, matrix = 256 × 256, voxel size = 1.13 × 1.13 × 1 mm^3^, FOV = 289 × 289 mm^2^). rs-fMRI data based on blood oxygen level-dependent (BOLD) contrast images with fat saturation were acquired using a gradient single-shot echo-planar imaging sequence with the following parameters; TE = minimum full (≈19 msec), flip angle = 90^o^, matrix = 64 × 64, FOV = 192 × 192 mm^2^, voxel size = 3 × 3 × 3 mm^3^, TR = 3,000 msec, 60 axial slices with no gap, and slice thickness of 3 mm. The scan duration was 9 min, allowing to collect 180 whole-brain volumes.

#### fMRI data preprocessing

Functional MRI data preprocessing was performed using SPM12 (http://www.fil.ion.ucl.ac.uk/spm) and the CONN toolbox (v.18b) (https://www.nitrc.org/projects/conn) was implemented in Matlab (R2018a) (https://uk.mathworks.com/products/matlab). Preprocessing steps followed CONN’s default pipeline and included motion estimation and correction by volume realignment using a six-parameter rigid body transformation, slice-time correction, and simultaneous gray matter (GM), white matter (WM), and cerebrospinal fluid (CSF) segmentation and normalization to MNI152 stereotactic space (2 mm isotropic) of both functional and structural data. Following preprocessing, the following potential confounding effects were removed from the BOLD signal using linear regression: six motion parameters calculated at the previous step and their first and second order derivatives, volumes with excessive movement (motion greater than 0.5 mm and global signal changes larger than z = 3), signal linear trend, and five principal components of the signal from WM and CSF (CompCor approach). Finally, data were band-pass filtered between 0.01 and 0.1 Hz.

#### fMRI functional connectivity analyses

Following preprocessing and denoising, we created two 6 mm radius spheres centered on the left and right pSTS (left pSTS: x = −55, y = −43, z = 5 and right pSTS: x = 55, y = −38, z = 4) taken from our previous TMS study of the bilateral pSTS (Sliwinska and Pitcher 2018). We then performed seed-to-voxel functional connectivity analyses in CONN, calculating spatial maps corresponding to the bivariate correlation (following HRF weighting) between the average time series within each seed and the rest of the brain, for each participant. These maps were entered into group-level analyses in order to (i) calculate the group mean connectivity maps of the two seeds separately, (ii) identify the brain regions where the connectivity to the left pSTS is greater than to the right pSTS and vice versa (contrasts left pSTS > right pSTS and right pSTS > left pSTS), and (iii) identify regions in these contrasts connected to pSTS in only one hemisphere. For (iii), we calculated z-maps of the individual seed maps produced in (i) and ran a formal conjunction analysis. The regions that survived a cluster z threshold of z = 3.1 and a cluster probability threshold of *P* = 0.01 were then removed from the results of analyses (ii). Mean framewise displacement and the number of data affected by motion (volumes with excessive movement as defined above) per participant were entered at the group level as confounds. A probabilistic 50% GM mask (cerebellum excluded) was applied to all group results, and they were FWE-corrected at *P* < 0.05.

#### Results

**Figure 5:**
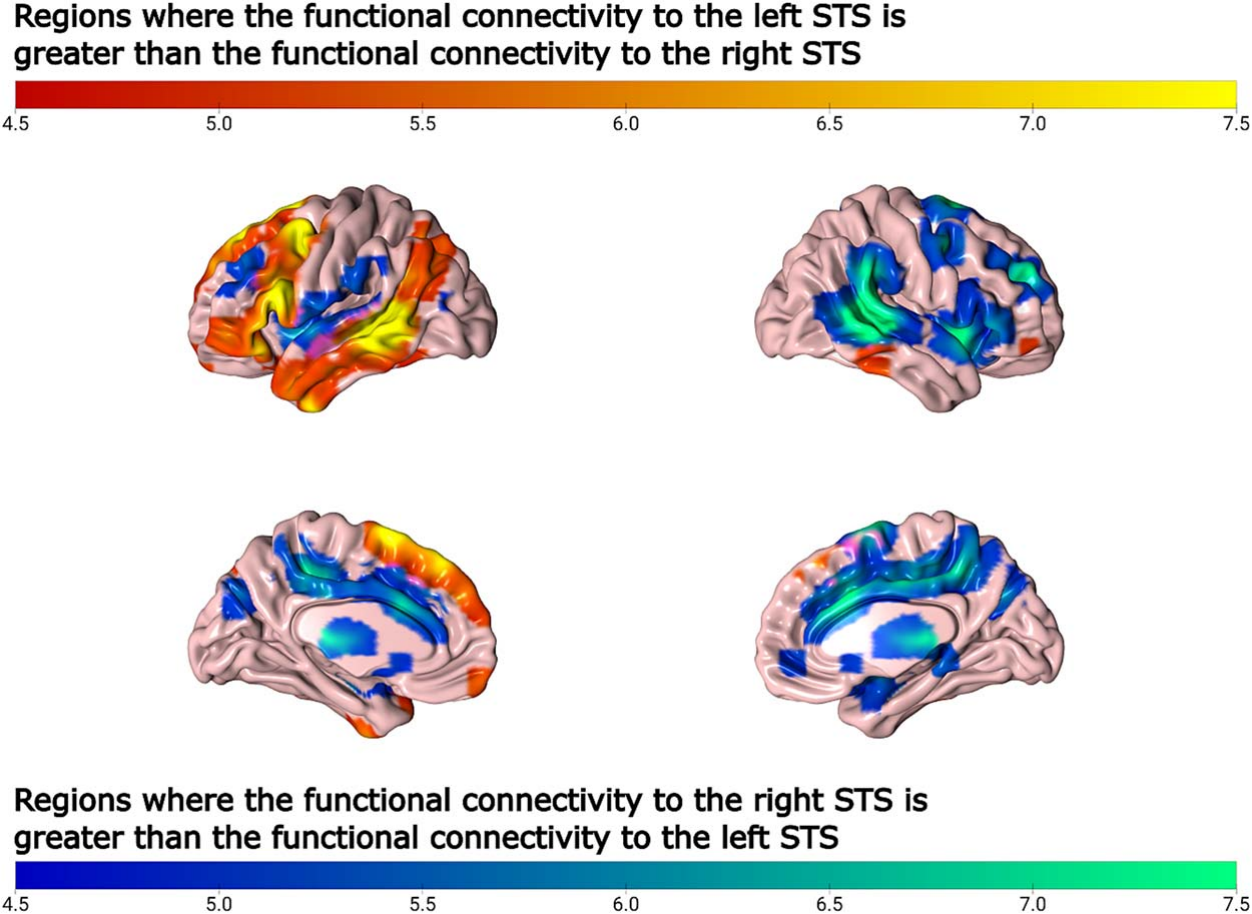
The connectivity maps from the first analysis. Regions where the functional connectivity to the left pSTS is greater than the functional connectivity to the right pSTS (red-yellow) and vice versa (blue-green).

The first analysis demonstrated regions showing greater functional connectivity to the left pSTS (red–yellow) than to the right pSTS, as well as regions showing greater connectivity to the right pSTS (blue–green) than to the left pSTS (Fig. 5). On the lateral surface of the left hemisphere (top left), the left pSTS was more strongly connected to the inferior and middle temporal gyrus, inferior parietal lobule and frontal regions, including the inferior frontal gyrus and the posterior part of the middle frontal gyrus. On the medial surface of the left hemisphere (bottom left), the left pSTS showed greater connectivity to the dorsomedial and ventromedial prefrontal cortex. In the right hemisphere (right images), the left pSTS was more connected to the anterior middle frontal gyrus and dorsomedial prefrontal cortex.

On the lateral surface of the left hemisphere (top left), the right pSTS was more connected to the temporo-parietal regions, including the superior temporal gyrus and superior parietal lobule, and to frontal regions including part of the middle frontal gyrus. On the medial surface (bottom left and right), the right pSTS showed greater connectivity to the anterior cingulate cortex, precuneus, caudate, medial parietal cortex, thalamus, amygdala, and cuneus bilaterally. On the lateral surface of the right hemisphere (top right image), the right pSTS was also connected more to the temporo-parietal regions, including posterior superior temporal gyrus, superior and inferior parietal lobule, and frontal regions including posterior and anterior parts of the middle frontal gyrus and inferior frontal gyrus.

**Figure 6:**
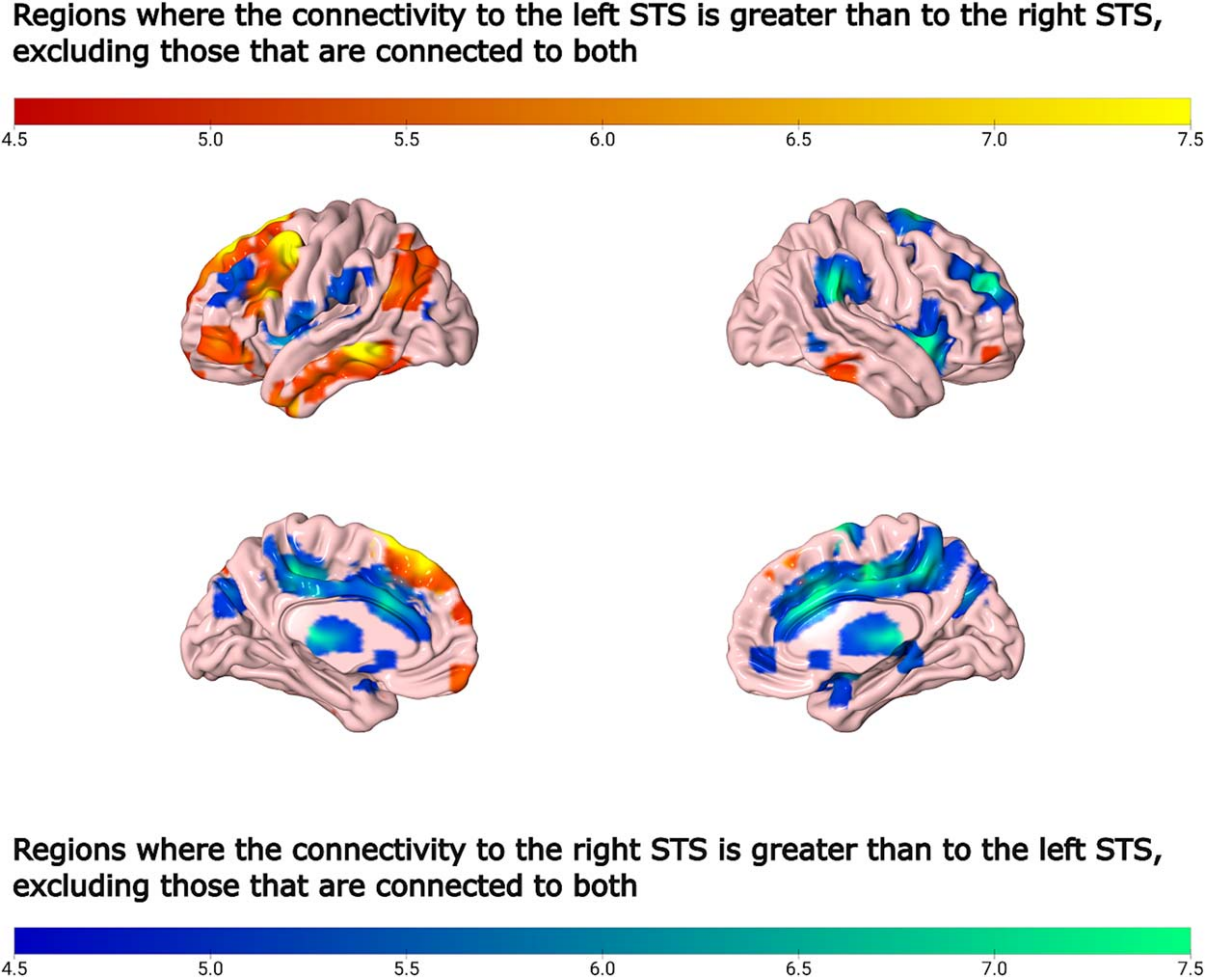
The connectivity maps from the second analysis. Regions where the functional connectivity to the left pSTS (red-yellow) is greater than the functional connectivity to the right pSTS, excluding those regions that are connected to both left and right pSTS, and vice versa (blue-green).

The second analysis identified regions where functional connectivity to the left pSTS (red–yellow) was greater than connectivity to the right pSTS, excluding regions connected to both the left and right pSTS, and regions where connectivity to the right pSTS (blue–green) was greater than connectivity to the left pSTS (Fig. 6). This analysis shows the connectivity of left pSTS is more lateralized than its right hemisphere homologue and encompasses regions of the left-lateralized language network, including speech-related regions in the classical Broca’s Area within the left inferior frontal region and the classical Wernicke’s Area within the temporo-parietal region (Binder et al. 1997; Price 2010). The connectivity pattern of the right pSTS was more bilateral and linked to attention and spatial processing (Corbetta et al. 2008; Shulman et al. 2009).

## Discussion

The present study investigated hemispheric specialization within the pSTS by combining causal brain stimulation, behavioral tasks, and resting-state functional connectivity analyses. Across three experiments, we demonstrated a functional dissociation between the left and right pSTS. The left pSTS was preferentially involved in processing speech-related visual information from the mouth, whereas the right pSTS was preferentially involved in processing eye-gaze direction. Experiment 1 was designed as a direct continuation of our previous TMS studies of the bilateral pSTS (Pitcher 2014; Sliwinska and Pitcher 2018; Sliwinska, Elson, and Pitcher 2020). By retaining the same delayed match-to-sample paradigm used in prior work but manipulating whether participants attended to eye-gaze or lip position, we were able to isolate the contribution of left and right pSTS to different facial cues within a task already known to depend causally on pSTS involvement. The resulting double dissociation demonstrates that hemispheric specialization within pSTS is not uniform across all facial information but depends on the social cue being processed. Experiment 2 extended these findings to dynamic, speech-related stimuli. Disruption of the left, but not the right, pSTS selectively impaired lip-reading performance, indicating that left pSTS is not sensitive to mouth features in general but is also preferentially involved in processing visually derived speech information. This result is consistent with prior neuropsychological and neuroimaging studies of audiovisual speech perception and the McGurk effect (eg Calvert and Campbell 2003; Beauchamp et al. 2010; De Winter 2015) and clarifies the functional role of left pSTS within the language network. Experiment 3 provided a network-level account of these causal dissociations. Resting-state connectivity analyses revealed that left pSTS is more strongly coupled with left-lateralized language regions, whereas right pSTS is embedded within more bilateral networks associated with visuospatial attention and social orienting. These connectivity profiles mirror the behavioral and TMS results and suggest that hemispheric specialization within the pSTS reflects differential integration into large-scale language and attention systems. Together, these findings support a model in which pSTS operates as a feature-sensitive hub for social perception, with hemispheric specialization emerging from the nature of the information being extracted and the networks to which each pSTS is connected. By building directly on our previous causal studies and integrating behavioral, stimulation, and connectivity evidence, the present work provides an account of how hemispheric specialization supports different components of human social perception.

Consistent with prior research, the results of Experiment 1 demonstrate that eye-gaze discrimination is preferentially processed in the right pSTS. Prior TMS and neuropsychological data demonstrated that disruption of the right pSTS impaired eye-gaze discrimination (Pourtois et al. 2004; Akiyama et al. 2006a; Akiyama et al. 2006b). However, these studies did not systematically test the causal role of the left pSTS. Our results show a clear dissociation between stimulation of the right pSTS, which impaired task performance, and stimulation of the left pSTS, which had no effect on task performance. While prior fMRI studies report a bilateral response in the pSTS to eye-gaze discrimination (Engell and Haxby 2007; Schobert et al. 2018), other studies show a greater neural response in the right pSTS (Hoffman and Haxby 2000). In addition, eye-gaze discrimination has been shown in the anterior STS of the right hemisphere in a study that used fMRI adaptation (Calder et al. 2007). This lateralized processing of eye-gaze discrimination is consistent with the established role of the right hemisphere in attention and spatial processing (Shulman et al. 2010). This right hemisphere bias is also reported in frontoparietal attention networks that link to brain areas involved in theory of mind and social cognition (Corbetta et al. 2008).

The results of Experiments 1 and 2 demonstrated that TMS delivered over the left, but not the right, pSTS impaired task performance on a static and dynamic lip-position matching task, respectively. This is in line with an fMRI study that investigated dynamic facial movements in lip-reading stimuli and demonstrated an increased activation in the superior temporal gyrus for participants with high and average lip-reading abilities, although this activation was shown to be bilateral (Ludman et al. 2000). Our study is also consistent with a prior TMS study that demonstrated that disruption of the left pSTS impaired the McGurk effect (Beauchamp et al. 2010). However, this study did not stimulate the right pSTS so no conclusions regarding laterality could be drawn. Intriguingly, the same group reported data from a neuropsychological patient with an extensive lesion to her left pSTS that exhibited a greater response to the McGurk effect in her right pSTS than in age-matched controls (Baum et al. 2012). The authors concluded this resulted from cortical reorganization subsequent to her incident. Our results from Experiment 2 clearly demonstrate that only disruption of the left pSTS, and not the right pSTS, impaired task performance. Cortical reorganization would not have occurred in our study because online TMS has no long-lasting effects on task performance.

Beyond the left pSTS, the audiovisual integration of speech is known to engage a distributed network of brain areas, including the upper and lower bank of the STS, the motor cortex, and the lateral prefrontal cortex (Bernstein and Liebenthal 2014). This is consistent with the results of Experiment 3 which showed greater functional connectivity between the left pSTS and lateral regions of the left frontal lobe and left anterior temporal lobe (in orange) that were not connected to the right pSTS (in blue) (Fig. 5). By contrast, the functional connectivity of the right pSTS was present in the lateral frontal and temporal lobes of both hemispheres, as well as on the bilateral medial surface of the brain (Fig. 6). This greater connectivity of the right pSTS is consistent with the dominant role of the right hemisphere in attention and spatial processing tasks (Corbetta et al. 2008; Shulman et al. 2009; Shulman et al. 2010). A right hemisphere preference in the pSTS for cognitive tasks reliant on unimodal visual input was also proposed in our third visual pathway model for visual social perception that projects from primary visual cortex via the motion-selective area V5/MT into the pSTS (Sliwinska, Bearpark, et al. 2020; Pitcher 2021; Pitcher and Ungerleider 2021).

It is worth exploring further why this pattern of lateralization emerges within the pSTS. One possibility is that different social cues depend on access to partially distinct perceptual and sensorimotor networks. Lip movements are visually perceived but are also closely linked to speech perception and audiovisual integration, processes that are typically left-lateralized and associated with language-related regions of the superior temporal cortex. Meta-analytic and neuroimaging evidence suggests that the left STS is strongly involved in speech perception and the integration of auditory and visual language information (Hocking and Price 2008; Liebenthal et al. 2014). In contrast, the perception of eye gaze is more closely associated with visuospatial attention, face perception, and the interpretation of socially relevant intentions. Functional imaging studies have consistently implicated the right pSTS in processing dynamic gaze cues and have shown that this region exhibits functional connectivity with other right-lateralized face- and attention-related regions, including the fusiform gyrus and anterior insula (Ethofer et al. 2011; Baseler et al. 2014). This organization may reflect a broader principle of hemispheric specialization in which the left hemisphere is biased toward language and audiovisual speech processing, whereas the right hemisphere is preferentially engaged in social attention and dynamic facial cue processing. Such functional segregation could support parallel processing and facilitate rapid access to socially relevant information during real-world interactions.

In conclusion, our study showed differential functional contributions of bilateral face-selective regions in pSTS. The left pSTS is preferentially involved in processing information from a person’s lips while the right pSTS is preferentially involved in processing information from a person’s eyes. This functional division is clearly supported by the differential brain connectivity patterns of those regions. This study provides additional evidence in support of the left hemisphere’s causal importance to facial recognition and illustrates a cross-functional importance in completing a face recognition task.
